# Prevalence and predictors of loss of wild type *BRCA1 *in estrogen receptor positive and negative *BRCA1*-associated breast cancers

**DOI:** 10.1186/bcr2776

**Published:** 2010-11-16

**Authors:** Nadine Tung, Alexander Miron, Stuart J Schnitt, Shiva Gautam, Katharina Fetten, Jennifer Kaplan, Yosuf Yassin, Ayodele Buraimoh, Ji-Young Kim, Attila M Szász, Ruiyang Tian, Zhigang C Wang, Laura C Collins, Jane Brock, Karen Krag, Robert D Legare, Dennis Sgroi, Paula D Ryan, Daniel P Silver, Judy E Garber, Andrea L Richardson

**Affiliations:** 1 Division of Hematology-Oncology, Beth Israel Deaconess Medical Center, Brookline Ave, Boston, MA 02215, USA; 2 Department of Cancer Biology, Dana-Farber Cancer Institute, Binney Street, Boston, MA 02115, USA; 3 Department of Pathology, Beth Israel Deaconess Medical Center, Brookline Ave, Boston, MA 02215, USA; 4 Department of Biostatistics Beth Israel Deaconess Medical Center, Brookline Ave, Boston, MA 02215, USA; 5 Department of Pathology, CHA Gangnam Medical Center, Yeoksamdong, Gangnamgu, Seoul, 135-081, Korea; 6 Program in Oncology, MGH/North Shore Cancer Center, Endicott Street, Danvers, CT 01923, USA; 7 Program in Women's Oncology, Women and Infants Hospital, Dudley St., Providence, RI 02905, USA; 8 Department of Pathology, Massachusetts General Hospital, Fruit St, Boston, MA 02114, USA; 9 Division of Medical Oncology, Massachusetts General Hospital, Fruit St, Boston, MA 02114, USA; 10 Division of Population Sciences and Adult Oncology, Dana-Farber Cancer Institute, Binney Street, Boston, MA 02115, USA; 11 Department of Pathology, Brigham and Women's Hospital, Francis Street, Boston, MA 02115, USA; 12 2nd Department of Pathology, Semmelweis University, Ulloi ut, Budapest, 01091, Hungary; 13 Department of Medical Oncology, Dana-Farber Cancer Institute, Binney Street, Boston, MA 02115, USA; 14 Harvard Medical School, Shattuck Street, Boston, MA 02115, USA

## Abstract

**Introduction:**

The majority of breast cancers that occur in *BRCA1 *mutation carriers (*BRCA1 *carriers) are estrogen receptor-negative (ER-). Therefore, it has been suggested that ER negativity is intrinsic to *BRCA1 *cancers and reflects the cell of origin of these tumors. However, approximately 20% of breast cancers that develop in *BRCA1 *carriers are ER-positive (ER+); these cancers are more likely to develop as *BRCA1 *carriers age, suggesting that they may be incidental and unrelated to *BRCA1 *deficiency. The purpose of this study was to compare the prevalence of loss of heterozygosity due to loss of wild type (wt) *BRCA1 *in ER+ and ER- breast cancers that have occurred in *BRCA1 *carriers and to determine whether age at diagnosis or any pathologic features or biomarkers predict for loss of wt *BRCA1 *in these breast cancers.

**Methods:**

Relative amounts of mutated and wt *BRCA1 *DNA were measured by quantitative polymerase chain reaction performed on laser capture microdissected cancer cells from 42 ER+ and 35 ER- invasive breast cancers that developed in *BRCA1 *carriers. *BRCA1 *gene methylation was determined on all cancers in which sufficient DNA was available. Immunostains for cytokeratins (CK) 5/6, 14, 8 and 18, epidermal growth factor receptor and p53 were performed on paraffin sections from tissue microarrays containing these cancers.

**Results:**

Loss of wt *BRCA1 *was equally frequent in ER+ and ER- *BRCA1*-associated cancers (81.0% vs 88.6%, respectively; *P *= 0.53). One of nine cancers tested that retained wt *BRCA1 *demonstrated *BRCA1 *gene methylation. Age at diagnosis was not significantly different between first invasive ER+ *BRCA1 *breast cancers with and without loss of wt *BRCA1 *(mean age 45.2 years vs 50.1 years, respectively; *P *= 0.51). ER+ *BRCA1 *cancers that retained wt *BRCA1 *were significantly more likely than those that lost wt *BRCA1 *to have a low mitotic rate (odds ratio (OR), 5.16; 95% CI, 1.91 to ∞). *BRCA1 *cancers with loss of wt *BRCA1 *were more likely to express basal cytokeratins CK 5/6 or 14 (OR 4.7; 95% CI, 1.85 to ∞).

**Conclusions:**

We found no difference in the prevalence of loss of wt *BRCA1 *between ER+ and ER- invasive *BRCA1*-associated breast cancers. Our findings suggest that many of the newer therapies for *BRCA1 *breast cancers designed to exploit the *BRCA1 *deficiency in these cancers may also be effective in ER+ cancers that develop in this population.

## Introduction

Sixty-four to 90% of breast cancers that occur in *BRCA1 *mutation carriers (*BRCA1 *carriers) are estrogen receptor-negative (ER-), progesterone receptor-negative (PR-) and lack HER2 protein overexpression and gene amplification, so called "triple negative" breast cancers [1-8]. These *BRCA1*-associated ER- tumors typically demonstrate characteristic pathologic features which include high grade ductal histology, a high mitotic rate, a prominent lymphocytic infiltrate, pushing or circumscribed margins, and geographic areas of necrosis or a central fibrotic focus [3,9,10]. In addition, these tumors often express "basal" biomarkers and cluster within the "basal-like" group in gene expression profiling studies [7,11-13].

Since *BRCA1 *cancers are so often ER-, it has been suggested that ER negativity is intrinsic to *BRCA1 *cancers and reflects the cell of origin of these tumors [14]. Preclinical models suggest that *BRCA1 *can transcriptionally induce ER gene expression and that loss of *BRCA1 *function is accompanied by loss of ER expression [15-17]. However, approximately 10 to 36% of breast cancers that occur in *BRCA1 *carriers are estrogen receptor-positive (ER+) [4,6,8,18,19]. Further, as *BRCA1 *carriers age, they are increasingly more likely to develop an ER+ breast cancer [14,20] following the trend seen in breast cancers that develop in the general population. It has, therefore, been suggested that ER+ *BRCA1*-associated breast cancers may actually be incidental or sporadic rather than caused by a complete loss of *BRCA1 *function.

We have previously shown that the pathologic features of ER+ invasive breast cancers that arise in *BRCA1 *carriers are significantly different than age-matched sporadic ER+ breast cancers in non-mutation carriers. When compared to sporadic ER+ cancers, ER+ *BRCA1*-associated cancers are more often of invasive ductal type and exhibit a high mitotic rate [20]. With the development of treatments such as poly(ADP-ribose) polymerase (PARP) inhibitors that are targeted to the specific defects in DNA repair pathways which exist in *BRCA1 *deficient cancers [21], it is important to determine whether ER+ breast cancers that develop in *BRCA1 *mutation carriers are incidental (that is, not directly related to the *BRCA1 *mutation/*BRCA1 *dysfunction) or if they are mutation-related in order to determine whether such *BRCA1*-targeted therapies might be effective in this population.

One way to address this issue is to analyze ER+ cancers that arise in *BRCA1 *mutation carriers for loss of the wild type (wt) *BRCA1 *allele. Several recent studies evaluating the prevalence of loss of heterozygosity (LOH) in *BRCA1*-associated breast cancers have noted that 50 to 90% of these cancers show LOH, with loss of wt *BRCA1 *[22-24]. However, none of these studies was designed to specifically evaluate loss of wt *BRCA1 *in relation to ER status in *BRCA1*-associated cancers. Therefore, we undertook a study to 1) determine the prevalence of loss of heterozygosity with loss of the wt *BRCA1 *allele in ER+ cancers from *BRCA1 *mutation carriers and compare it to that found in ER- *BRCA1-*associated cancers; and 2) determine whether any clinical factors (that is, age at diagnosis), pathologic features or biomarkers predict for loss of wt *BRCA1 *in *BRCA1*-associated breast cancers.

## Materials and methods

A series of 51 ER+ and 47 ER- invasive breast cancers was assembled from 88 women with deleterious *BRCA1 *germ-line mutations who had undergone genetic testing at five high risk genetic programs.

Age at diagnosis of the breast cancer and determination of whether the cancer was a first or subsequent cancer for the patient was determined from medical record review. Specific *BRCA1 *mutations were confirmed by review of genetic test reports.

Histologic sections of *BRCA1*-associated ER- and ER+ breast cancers were reviewed by the study pathologists blinded to the ER status of the tumor prior to the determination of LOH status. Each cancer was scored for the following pathologic features: histologic type; Nottingham combined histologic grade, with each of the three components of grade (that is, tubule formation, nuclear grade and mitotic rate) recorded separately; presence of geographic necrosis or fibrotic focus; extent of lymphocytic infiltrate; and tumor margin characteristics (invasive or pushing).

Information regarding ER, PR and HER2 status, assessed as part of the routine clinical evaluation, was abstracted from institutional pathology reports. When information regarding the ER, PR or HER2 status for *BRCA1 *tumors was missing from the pathology report, paraffin blocks were re-cut and sections were immunostained for ER (SP1 antibody, Neomarkers, Fremont, CA, USA), PR (PgR 636 antibody, Dako, Carpinteria, CA, USA) and HER2 (A085 antibody, Dako). For ER- tumors, ER expression was re-evaluated if the pathology report did not state that an appropriately staining internal positive control was present. Similarly, ER+ tumors with reports stating "faint" or "low" ER staining were re-evaluated for ER expression. If greater than 1% of breast cancer cells stained for ER, the tumor was considered ER+. If fluorescence *in situ *hybridization (FISH) for HER2 was available for a cancer, this result was used rather than the IHC result.

Tissue microarrays were constructed by obtaining three 0.6 mm cores from the formalin-fixed paraffin embedded blocks of each *BRCA1 *cancer and placing them in recipient paraffin blocks. Slides cut from the TMAs were immunostained for cytokeratin (CK) 5/6 (D5/16B4 antibody, Dako), CK14 (LL02 antibody, Neomarkers), CK7/8 (Cam5.2 antibody, BD Biosciences, San Jose, CA, USA), CK18 (DC10 antibody, Dako) and CK19 (RCK108 antibody, Dako) as well as for EGFR (EGFR pharmDX kit, Dako) and p53 (DO-7 antibody, Dako).

This study was approved by the institutional review boards of Dana Farber/Harvard Cancer Center and North Shore Medical Center.

### Assessment of *BRCA1 *LOH

Representative slides from each cancer were reviewed by a study pathologist and areas of normal tissue as well as invasive cancer were identified. Cells were isolated from hematoxylin and eosin stained tissue sections by laser capture microdissection. Approximately 2,000 pulses were used for each microdissection. As higher levels of normal cell contamination will reduce the proportion of mutant allele that is measured in a tumor with true LOH, for each tumor we attempted at least two microdissections performed at the same general region. For each case, an area of normal non-tumor tissue present in the same block as the cancer was microdissected to obtain germline DNA. The microdissected cells were lysed to release DNA by overnight digestion with proteinase K, followed by a second round of proteinase K digestion, and then heat inactivation was performed.

Screening for LOH was carried out by polymerase chain reaction (PCR) followed by Sanger dideoxy sequencing [25] or missense and nonsense alterations, while denaturing capillary analysis was performed for insertions and deletions. Exon specific oligonucleotide primers flanking known *BRCA1 *mutations were designed using Primer3 software [26] and limited to a maximum product size of 200 base pairs in order to successfully amplify sheared DNA extracted from paraffin embedded tissue (sequences listed in Additional file 1). PCR primers were ordered [27] with universal sequence tags at the 5' end in order to allow for secondary amplification, sequencing with a universal primer and elimination of primer dimers.

For cancers with missense and nonsense mutations, bidirectional DNA sequencing in triplicate was performed using BigDye Terminator v3.1 Cycle Sequencing Kit (Applied Biosystems, Foster City, CA, USA) and universal primers. Purified sequencing products were analyzed in 3730xl DNA Analyzers (Applied Biosystems). Sequence data from triplicate forward and reverse reactions from each micro-dissected tumor and normal surrounding tissue were analyzed using the Mutation Surveyor program version 3.30 (SoftGenetics, State College, PA, USA). The NM_Score is a value assigned to each nucleotide in a sequencing chromatogram. This score is used to determine mutant allele contribution based on shape and height at each position in the sequence. Normal (that is, non-cancerous) tissue DNA from *BRCA1 *carriers is generally heterozygous for *BRCA1 *mutation, and therefore sequence NM_scores are less than 1.5 indicating a significant difference from homozygous wild-type. In tumors with LOH and loss of the wild type allele, the difference from homozygous wild-type becomes even more significant as the NM_score decreases further towards 0.

Analysis of LOH in tumors with insertions or deletions in *BRCA1 *was performed by taking advantage of the difference in size of the wild type and mutant alleles. Primary amplification was performed using the same conditions described for sequencing analysis above. Secondary PCR amplification was used to add a 6-FAM fluorescent label onto the 5'-end of the primary PCR products. Reaction products were then analyzed on ABI3730XL (Applied Biosystems) instruments using POP7 denaturing polymer (Applied Biosystems). The relative height of the wild type and mutant peaks was measured using Dax software (Van Mierlo Software Consultancy, Eindhoven, NL) and was used to indicate LOH. An example of the data for 187delAG analysis is shown in Additional file 2. Further details of the methodology used for LOH determination are included in Additional file 3[28-30].

### Analysis of *BRCA1 *promoter methylation

Tumor DNA was subjected to bisulfite modification using EZ DNA methylation-gold kit (Zymo Research, Orange, CA, USA). Lymphocyte DNA treated with Sss bacterial methylase (New England BioLabs, Ipswich, MA, USA) was used as a positive control. Lymphocyte DNA treated with bisulfite served as a negative control. Bisulfite treated tumor DNAs were analyzed by methylation specific PCR for *BRCA1 *promoter as in Matros *et al*. [31]. The methylation assay evaluates CpG sites within the *BRCA1 *promoter at positions -37, -29, -21, -19 from the start site. PCR products were resolved by 3% agarose gel electrophoresis.

### Statistics

#### Determination of cutoff values for LOH

##### Tumors with insertion/deletion mutations

The percentage of mutant *BRCA1 *DNA (mutant *BRCA1 *DNA/mutant plus wt *BRCA1 *DNA; m%) was determined by comparing peak heights specific for normal and mutant alleles determined from DNA extracted from each microdissected sample of normal tissue and tumor tissue. PCR reactions were run in triplicate for each microdissected normal and tumor tissue sample and those samples with three successful amplifications were retained for further consideration. Coefficients of variation (CV) of the triplicate m% and 100-m% values were calculated for each microdissected sample and those with a CV greater than 20% were not considered further. For each microdissected sample, the average m% from the triplicate PCR reactions was determined. If multiple microdissected samples from a single tumor all showed acceptably low m% CV's then the results from the separate microdissections were averaged to give a single average m% for that tumor or normal specimen. Determination of LOH for *BRCA1 *was guided by the assumption that the majority of normal tissue specimens should not have LOH, though there are data that some lobules from normal appearing breast tissue obtained from *BRCA1 *carriers undergoing prophylactic mastectomies can have LOH with loss of wt *BRCA1 *[32]. Two different strategies were adopted to determine the optimal m% cut-off for a determination of LOH. First, logistic regression was used to optimally discriminate between tumor and normal tissue yielding cut-off values for LOH. For this computation all specimens were assumed to be independent. In the second approach, the range of average m% values was determined for all the adjacent normal tissues dissected from tumors with insertion or deletion mutations. The 95% CI for average m% for normal tissue was determined within two standards of deviations. Tumors with an average m% above the upper limits of this 95% CI were determined to have LOH with loss of wt *BRCA1 *and tumors with average m% below the limits of the 95% CI were determined to have LOH with loss of the mutant *BRCA1 *allele (LOH^mut^). Both methods yielded virtually identical results. An average m% > 60 was considered to represent LOH with loss of wt *BRCA1 *and m% < 40 was considered to represent LOH^mut ^in the 59 cancers with small insertions or deletions.

The 1294del40 mutation results in a 40 bp deletion. The significant difference in size between the PCR products from the normal allele vs. the deletion mutant allele results in preferential amplification of the smaller mutant allele and a skewed ratio of mutant to normal PCR products. Therefore, the determination of LOH cut-off was performed separately for the 1294del40 mutations using the same process described above considering both the distribution of m% in normal 1294del40 heterozygous tissues as well as insight from logistic regression. An average m% of > 85 was established as an appropriate cut-off for determining loss of wt *BRCA1 *for the 1294del40 cases.

##### Tumors with missense and nonsense mutations

For cases with missense and nonsense mutations, the NM_Score (generated by the Mutation Surveyor program) was used to determine LOH. NM_scores from the forward sequencing triplicate data and reverse sequencing triplicate data were averaged separately for each case due to base and strand specific variation in the score. Samples with forward and reverse triplicate NM_score measurements showing a CV < 20% were included. Multiple microdissected samples from a single tumor with acceptably low NM_score CV's were averaged to give a single average NM_score for that case. The same process described above using logistic regression as well as 95% CI for normal tissue NM_score was used to determine the LOH cutoff for the 14 cancers with missense or nonsense mutations. An NM_score of < 0.6 was deemed to represent LOH with loss of wt *BRCA1 *and NM_score > 1.4 was interpreted as LOH^mut^.

#### Predictors of loss of wt *BRCA1*

The Wilcoxon Rank Sum test was used to determine *P*-value differences in mean age between *BRCA1 *first invasive breast cancers with and without loss of wt *BRCA1*. The two-sided Fisher exact test was used to determine differences in the frequency of variables between cancers with and without loss of wt *BRCA1 *on univariate analysis. The Bonferroni calculation identified *P *< 0.003 as significant after correction for multiple comparisons. In the multivariate analyses, exact logistic regression was used to evaluate the association between loss of wt *BRCA1 *and the variables found significant in the univariate analyses. For these analyses, the cases with LOH and loss of wt *BRCA1 *were compared to the combined group of cases showing either no LOH or LOH^mut^.

## Results

### Prevalence of loss of wt *BRCA1*

Ninety-eight *BRCA1*-associated ER+ and ER- invasive breast cancers were used for this analysis. Of the 98 ER+ and ER- breast cancers from women with *BRCA1 *mutations, the status of the wt *BRCA1 *allele could not be obtained for 21 cancers (12 ER- and 9 ER+): in 4 cancers (1 ER- and 3 ER+), microdissection for isolation of a pure population of tumor cells was not feasible due to the intimate admixture of single tumor cells and normal cells; in 10 cancers (8 ER- and 2 ER+) PCR failed; in 7 cancers (3 ER- and 4 ER+) the results from triplicate PCR were too variable (that is, CV > 20%) for inclusion in analysis.

Thus, reliable determination of the status of the wt *BRCA1 *allele was possible in 77 *BRCA1*-associated breast cancers (42 ER+, 35 ER-). Of these cancers 34/42 (81.0%) ER+ and 31/35 (88.6%) ER- had LOH with loss of the wt *BRCA1 *allele; the difference in frequency of loss of wt *BRCA1 *between ER+ and ER- cancers was not significant (*P *= 0.53).

Six cancers (three ER+ and three ER-) with insertion/deletion mutations had m% < 40% and one cancer (ER+) with a point mutation had an NM score > 1.4 consistent with LOH with loss of the mutant *BRCA1 *allele. In sporadic breast cancers, particularly in ER- cancers, loss of heterozygosity involving large regions of chromosome 17, including the *BRCA1 *locus, is seen in 49 to 57% of cases [33-35]. In the *BRCA1*-associated cancers in this study, LOH with loss of either the wt or mutant *BRCA1 *allele was observed in 97% of ER- and 90% of ER+ cases. If LOH involving this region was unrelated to the *BRCA1 *mutation, loss of the wt and mutant alleles would be expected to occur with equal frequencies. As shown in Additional file 4, the observed frequencies of loss of the wt and mutant alleles, determined by chi-square test, are significantly skewed toward loss of wt *BRCA1 *(*P *< 0.001). There was no relationship between ER status and distribution of which allele was lost by LOH (*P *= 0.81). This result demonstrates the non-random relationship between LOH and selection for loss of wt *BRCA1 *in both ER+ and ER- *BRCA1*-associated tumors.

**Figure 1:**
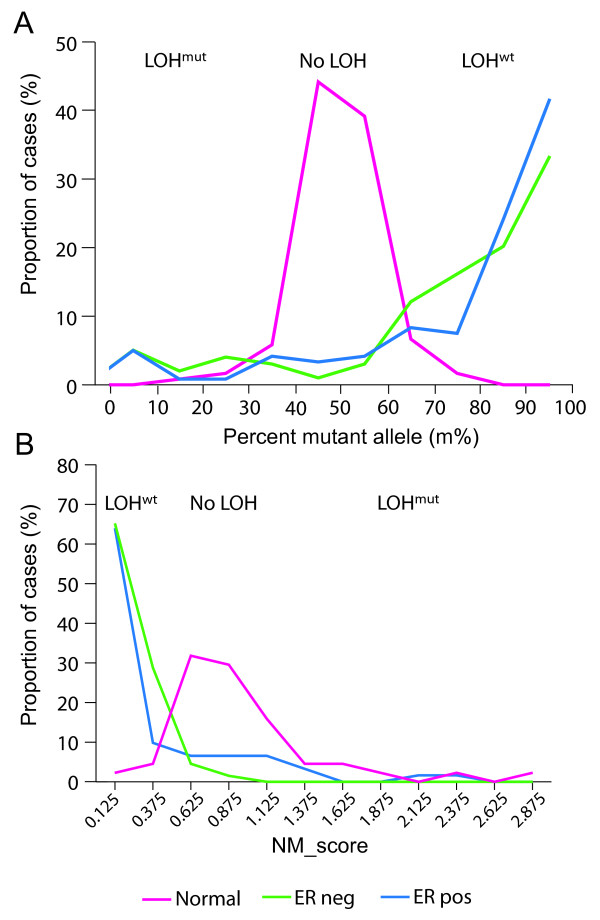
**The distributions of the percent mutant allele are similar for ER+ and ER- *BRCA1*-associated breast cancers**. The frequency distributions of percent mutant allele (m% values) for cases with insertion or deletion mutations in *BRCA1 *but excluding 1294 del40 cases **(a) **and the frequency distributions of NM_scores for cases with missense or nonsense point mutations **(b) **are shown for adjacent normal tissue samples (pink), ER+ tumor samples (blue), and ER- tumor samples (green). Distributions are calculated using all triplicate measurements from those samples passing quality assessment (that is, CV < 20%). In panel A, higher m% values indicate LOH with loss of wt *BRCA1 *allele (LOH^wt^) and lower values indicate LOH with loss of mutant *BRCA1 *allele (LOH^mut^). For panel B, lower NM_scores indicate loss of wt *BRCA1 *and higher NM_scores indicate loss of mutant *BRCA1*.

The distribution of m% values for normal and tumor samples (both ER+ and ER-) from insertion/deletion analysis are shown in Figure 1a. The mean and standard deviation of percent mutant allele from analysis of normal tissues was 50% ± 8.3%, indicating the expected proportion of mutant and wt alleles in heterozygous samples. The distribution of retained NM_scores from normal and ER+/ER- tumor samples with missense or nonsense mutation are shown in Figure 1b. The distributions for m% and NM_score for ER+ and ER- *BRCA1*-associated cancers were roughly similar. Several cases (seven ER- and two ER+) were noted to have an m% between 60 and 80%. Assuming a pure tumor sample, this intermediate level of mutant allele may indicate intratumoral heterogeneity for LOH, with an admixture of tumor cells with and without LOH. Alternatively, intermediate m% values may represent samples with slightly more normal cell contamination as an absolutely pure tumor sample is not always attainable, even with microdissection. Without performing an *in situ *LOH assay, it is not possible to distinguish these alternative explanations for any differences in distribution observed between ER+ and ER- cancers.

While the spectrum of *BRCA1 *mutations was varied in this group of 77 cancers, 57% of the mutations were Ashkenazi mutations (Additional file 5) reflecting the significant Jewish population in the communities served by the participating hospitals. Fifty-nine cancers occurred in women with a small *BRCA1 *insertion or deletion mutation, four additional women had a large 40 base-pair deletion (1294del40), seven had nonsense point mutations and three had a splice site mutation resulting in an in-frame deletion of an exon. Only four women had missense point mutations. Of the 12 cancers without loss of wt *BRCA1*, 10 (4 ER-, 6 ER+) had protein truncating lesions and 2 (both ER+) had a splice site mutation resulting in an in-frame deletion (Additional file 5). Specific mutations for each cancer as well as the percentage of mutated *BRCA1 *DNA (m%), NM_score, and ER status for each cancer is shown in Additional file 5.

### Clinical, pathologic and immunohistochemical findings

#### All *BRCA1*-associated breast cancers (ER+ and ER- combined)

When pathologic features and biomarkers expression of all *BRCA1*-associated cancers (ER+ and ER- combined) were analyzed according to status of loss of wt *BRCA1 *allele, in univariate analysis, breast cancers with loss of wt *BRCA1 *were more likely to be of pure invasive ductal type (*P *= 0.043), to be histologic grade 3 (*P *= 0.001), and to have a higher mitotic rate (*P *< 0.001) (Table 1). Cancers with loss of wt *BRCA1 *were also more likely to exhibit expression for CK5/6 (*P *< 0.001), CK14 (*P *= 0.023) or either CK5/6 or CK14 (*P *< 0.001) (Table 1). Cancers with loss of wt *BRCA1 *were more likely to express either CK5/6 or CK14 even after Bonferroni adjustment for multiple comparisons and in multinomial logistic regression (odds ratio (OR), 4.7; 95% CI, 1.85 to ∞).

**Table 1:** Pathologic and immunohistochemical characteristics that were significantly different in BRCA1-associated breast cancers with and without loss of wt BRCA1

Variables	Loss of wt *BRCA1*	No loss of wt *BRCA1*	*P*-value
	N(%)	N(%)	
**Sample Size**	65	12	
**Histology**			
Ductal*	59 (90.8)	8 (66.7)	**0.043**
Ductal/Lobular	4 (6.1)	3 (25)	
Other	2 (3.1)	1 (8.3)	
**Mitoses/10 high power field**			
0 to 5*	10 (15.4)	8 (66.7)	< 0.001*
6 to 10	14 (21.5)	0	
> 10	41 (63.1)	4 (33.3)	
**Grade**			
1 (3 to 5 points)	5 (7.7)	5 (41.7)	
2 (6 to 7 points)	7 (10.8)	3 (25)	
3 (8 to 9 points)*	53 (81.5)	4 (33.3)	0.001*
**CK5/6**	58	11	
Positive	29 (50)	0 (0)	< 0.001*
Negative	29 (50)	11 (100)	
**CK14**	58	9	**0.023**
Positive	23 (39.7)	0	
Negative	35 (60.3)	9 (100)	
**CK5/6 or CK14**	60	12	< 0.001*
Positive	35 (58.3)	0 ((0)	
Negative	25 (41.7)	12 (100)	
**CK5/6 and/or CK14 and/or EGFR**	61	12	**0.023**
Positive	39 (63.9)	3 (25)	
Negative	22 (36.1)	9 (75)	

Abbreviations: ER, estrogen receptor; CK, cytokeratin; EGFR, epidermal growth factor receptor.

Loss of wt: cancers with LOH with loss of wt *BRCA1 *allele.

No loss of wt: includes cancers with no LOH and LOH^mut^.

*P-value retained significance with Bonferroni adjustment for multiple comparisons.

Percentages calculated by column.

HER2 protein overexpression and/or gene amplification was found in four of the 77 *BRCA1*-associated breast cancers (one by IHC; three by FISH). Two of the three HER2 FISH amplified tumors did not show loss of wt *BRCA1*. The HER2 overexpressing tumor identified by IHC did demonstrate LOH for wt *BRCA1*; however, material was not available to analyze this case for HER2 gene amplification by FISH.

We also compared the frequency of pathologic features and biomarker expression of the 12 ER- and 9 ER+ *BRCA1*-associated cancers for which wt *BRCA1 *allele status could not be determined to ensure that this group was similar to those cancers which were successfully subjected to LOH analysis. No significant differences in any of the pathologic features or biomarkers were identified between the groups with and without successful *BRCA1 *LOH analysis.

#### ER+ *BRCA1*-associated cancers

The age at which first ER+ breast cancers developed was analyzed according to wt *BRCA1 *allele status. The mean age at diagnosis was 45.2 years for the 24 ER+ first breast cancers with loss of wt *BRCA1*, compared to 50.1 years in those 8 ER+ first cancers that retained a wt *BRCA1 *allele. This difference was not statistically significant (*P *= 0.51) (Table 2).

**Table 2:** Pathologic characteristics and their association with loss of wt BRCA1 in ER-positive and ER-negative BRCA1-related breast cancers

Variable	ER+			ER-		
		
	Loss of wt *BRCA1* N(%)	No loss of wt *BRCA1* N(%)	*P*-value	Loss of wt *BRCA1 *N(%)	No loss of wt *BRCA1* N(%)	*P*-value
**Sample Size**	34	8		31	4	
**First cancers**	24 (70.6)	8 (100)		28 (90.3)	2 (50)	
**Mean age (range)**	45.2 (29 to 68)	50.1 (33 to 72)	0.51	42 (28 to 73)	33.5 (31 to 36)	0.14
**Second cancers**	10 (29.4)	0		3 (9.7)	2 (50)	
**Mean age (range)**	48.7 (34 to 63)			56 (53 to 61)	57 (56 to 58)	
**Histology**						
Ductal*	29 (85.3)	4 (50)	**0.05**	30 (96.8)	4 (100)	1.00
Ductal/Lobular	3 (8.8)	3 (37.5)		1 (3.2)	0	
Other	2 (5.9)	1 (12.5)		0	0	
**Mitoses/10 high power field**						
0 to 5*	9 (26.5)	8 (100)	< 0.001*	1 (3.2)	0	1.00
6 to 10	12 (35.3)	0		2 (6.5)	0	
> 10	13 (38.2)	0		28 (90.3)	4 (100)	
**Grade**						
1 (3 to 5 points)	5 (14.7)	5 (62.5)		0	0	
2 (6 to 7 points)	6 (17.6)	3(37.5)		1 (3.2)	0	
3 (8 to 9 points)*	23 (67.6)	0	< 0.001*	30 (96.8)	4 (100)	1.00
**Geographic Necrosis/Fibrotic Focus**						
Yes	9 (26.5)	0	1.00	22 (71.0)	2 (50)	0.574
No	25 (73.5)	8 (100)		9 (29.0)	2 (50)	
**Margins**						
Invasive	29 (85.3)	8 (100)	1.00	15 (48.4)	2 (50)	1.00
Pushing/Circumscribed	5 (14.7)	0		16 (51.6)	2 (50)	
**Lymphocyte Infiltrate**						
Moderate/Severe	4 (11.8)	0	1.00	13 (41.9)	2 (50)	1.00
None/Mild	30 (88.2)	8 (100)		18 (58.1)	2 (50)	
**PR**						
Positive*	29 (85.3)	8 (100)	1.00	1 (3.6)	0	1.00
Positive	26 (76.5)	7 (87.5)		0		
Low Positive^a^	3 (8.8)	1 (12.5)		1 (3.6)		
Negative	5 (14.7)	0		27 (96.4)	4 (100)	
Unknown	0	0		3 (9.8)	0	
**Her2/Neu IHC**						
Positive	1 (2.9)	0	1.00	0	0	1.00
Negative	24 (70.6)	7 (87.5)		25 (80.6)	3 (75)	
**Her2/Neu FISH**						
Positive	1 (2.9)	1 (12.5)		0	1 (25)	
Negative	6 (17.6)	0		5 (16.1)	0	
Equivocal^d ^	2 (5.9)	0		1 (3.2)	0	

Abbreviations: ER, estrogen receptor; PR, progesterone receptor; IHC, immunohistochemistry; FISH, fluorescence *in situ *hybridization.

Loss of wt: cancers with LOH with loss of wt *BRCA1 *allele.

No loss of wt: includes cancers with no LOH and LOH^mut ^.

Percentages calculated by column.

^a ^Low PR positive: 1 to 10% cells show nuclear staining for PR.

^b ^HER2 positive: HER2:CEP17 ratio by FISH > 2.2 or IHC 3+ (no FISH performed).

^c ^HER2 negative: HER2:CEP17 ratio by FISH < 1.8 or IHC < 3+ (no FISH performed).

^d ^HER2 equivocal: HER2:CEP17 ratio by FISH 1.8 to 2.2.

* *P*-value retained significance with Bonferroni adjustment for multiple comparisons.

The pathologic features of the ER+ *BRCA1 *cancers with (*n *= 34) and without (*n *= 8) loss of wt *BRCA1 *are compared in Table 2. In univariate analysis, those ER+ *BRCA1 *cancers retaining wt *BRCA1 *more often had a low mitotic rate (100% vs 27%; *P *< 0.001), were less often of pure ductal histology (50% vs 85%; *P *= 0.05), and less often grade 3 (0% vs 68%; *P *< 0.001). The differences in mitotic rate and grade retained significance even after Bonferroni adjustment. In multinomial logistic regression, ER+ *BRCA1*-associated cancers retaining wt *BRCA1 *were significantly more likely than cancers without wt *BRCA1 *to have a low mitotic rate (OR, 5.16; 95% CI, 1.91 to ∞).

Results of biomarker studies were available for most of the *BRCA1*-associated cancers (Table 3). None of the eight ER+ cancers that retained wt *BRCA1 *showed expression of basal cytokeratins CK5/6 or CK14. In contrast, 11 of 33 ER+ cancers with loss of wt *BRCA1 *(33.3%) showed expression for one or the other of these basal cytokeratins. However, this difference was not statistically significant, possibly due to the small number of cases.

**Table 3:** Immunohistochemical characteristics and their association with loss of wt BRCA1 in ER-positive and ER-negative BRCA1-related breast cancers

Variable	ER+			ER-		
		
	Loss of wt *BRCA1 *N(%)	No loss of wt *BRCA1 *N(%)	*P*-value	Loss of wt *BRCA1 *N(%)	No loss of wt *BRCA1* N(%)	*P*-value
**Cytokeratins**						

**CK5/6**	(31)	(7)	0.164	(27)	(4)	**0.010**
Positive	9 (29.0)	0		20 (74.1)	0	
Negative	22 (71.0)	7 (100)		7 (25.9)	4 (100)	
**CK7/8**	(31)	(8)	---	(28)	(4)	1.0
Positive	31 (100)	8 (100)		25 (89.3)	4 (100)	
Negative	0	0		3 (10.7)	0	
**CK14**	(31)	(6)	0.566	(27)	(3)	0.054
Positive	5 (16.1)	0		18 (66.7)	0	
Negative	26 (83.9)	6 (100)		9 (33.3)	3(100)	
**CK5/6 or CK14**	(33)	(8)	0.083	(27)	(4)	0.001*
Positive	11 (33.3)	0		24 (88.9)	0	
Negative	22 (66.7)	8 (100)		3 (11.1)	4 (100)	
**CK 18**	(32)	(7)	----	(27)	(4)	1.00
Positive	32 (100)	7 (100)		26 (96.3)	4 (100)	
Negative	0	0		1 (3.7)	0	
**CK19**	(33)	(6)	----	(28)	(4)	0.125
Positive	33 (100)	6 (100)		28 (100)	3 (75)	
Negative	0	0		0	1 (25)	
**EGFR**	(30)	(7)	0.560	(26)	(4)	0.360
Positive	5 (16.7)	0		24 (92.3)	3(75)	
Negative	25 (83.3)	7 (100)		2 (7.7)	1 (25)	
**CK5/6 and/or CK14 and/or EGFR**	(33)	(8)	0.083	(28)	(4)	0.125
Positive	11 (33.3)	0		28 (100)	3 (75)	
Negative	22 (66.7)	8 (100)		0	1 (25)	
**P53**	(30)	(7)	1.00	(26)	(4)	0.601
Positive	14 (46.7)	4 (57.1)		13 (50)	3 (75)	
Negative	16 (53.3)	3 (42.9)		13 (50)	1 (25)	

Abbreviations: ER, estrogen receptor; CK, cytokeratin; EGFR, epidermal growth factor receptor.

Loss of wt: cancers with LOH with loss of wt *BRCA1 *allele.

No loss of wt: includes cancers with no LOH and LOH^mut ^.

*P-value retained significance with Bonferroni adjustment for multiple comparisons.

Percentages calculated by column.

#### ER- *BRCA1*-associated cancers

In ER- cancers, the mean age at diagnosis of first invasive cancers with or without loss of wt *BRCA1 *was 42 years and 33.5 years, respectively. This difference was not statistically significant (*P *= 0.15) (Table 2).

No significant histopathologic differences were noted between the 31 ER- cancers with loss of wt *BRCA1 *and the 4 ER- cancers with retention of wt allele. Like the ER- cancers with loss of wt *BRCA1*, the four ER- cancers retaining wt *BRCA1 *were high grade ductal cancers with a high mitotic rate. There were also no significant differences between tumors with or without loss of wt *BRCA1 *with regard to those features considered to be characteristic of ER- *BRCA1*-associated cancers, including geographic necrosis, pushing margins, and moderate-marked lymphocytic infiltrate (Table 2).

There were significant differences in the frequency of expression of basal cytokeratins CK5/6 and CK14 in ER- cancers with and without loss of wt *BRCA1 *in univariate analysis (Table 3). Expression of either CK5/6 or CK14 was significantly more frequent in the 27 ER- cases with loss of wt *BRCA1 *compared to the 4 cases without (89% vs. 0%, *P *= 0.001). This difference was significant even after Bonferroni correction. In multinomial logistic regression, ER- cancers with loss of wt *BRCA1 *were significantly more likely to show expression of CK5/6 or CK14 (OR 5.48; 95% CI 1.79 to ∞). There were no significant differences in EGFR or p53 expression between ER- cancers with and without loss of wt *BRCA1 *(Table 3).

#### *BRCA1* methylation

*BRCA1 *promoter methylation is a potential alternative mechanism to LOH for providing the "second hit" to inactivate wt *BRCA1 *in *BRCA1*-associated cancers. *BRCA1 *promoter methylation analysis was performed by methylation-specific PCR on 28 cases for which remaining tumor DNA was available, including 6 of the 8 ER+ and 3 of the 4 ER- cancers which did not demonstrate loss of wt *BRCA1*. Methylation was identified in a single tumor sample, an ER+ low-grade (tubular) carcinoma without genomic loss of wt *BRCA1*.

## Discussion

In this study, we found that 81.0% of ER+ *BRCA1*-associated breast cancers showed LOH with loss of the wt *BRCA1 *allele. The prevalence of loss of wt *BRCA1 *in these ER+ tumors was similar to that seen in the ER- *BRCA1*-associated cancers (88.6%). This is the first study, to our knowledge, that has specifically examined loss of wt *BRCA1 *in a large cohort of *BRCA1*-associated breast cancers in relation to ER status. Our results are consistent with those reported in previous smaller studies [22-24]. Only two of these prior studies included any ER+ *BRCA1 *cancers and reported loss of wt *BRCA1 *in 75% and 83% of such cancers [22,24].

Only one cancer of the 28 evaluated in our study demonstrated *BRCA1 *promoter methylation, an ER+ cancer with retention of wt *BRCA1*. These results are consistent with the lower cumulative methylation observed in *BRCA1*-associated cancers compared to sporadic cancers [36]. Our results are also comparable to those of Dworkin *et al*. who found that none of seven of their *BRCA1 *cancers without LOH showed methylation as a "second hit" [22]. The one ER+ cancer demonstrating *BRCA1 *methylation in our series was not typical of the ER+ cancers with loss of wt *BRCA1*, being a low grade (tubular) carcinoma with a low mitotic rate. Studies of sporadic breast cancers have not found reproducible associations between *BRCA1 *promoter methylation and tumor phenotype [31,37]. Furthermore, somatic methylation may be related to increasing age in some cases [36,38]. *BRCA1 *promoter methylation has been found in germline DNA in five to seven percent of individuals regardless of health or *BRCA1/2 *status and showed no association with development of breast cancer [39]. Whether the *BRCA1 *promoter methylation found in the tumor DNA of the one patient in our series is indicative of loss of wt *BRCA1 *function is uncertain and its clinical significance is unclear.

Previously, we have described that ER+ *BRCA1*-associated cancers are more often high grade ductal cancers compared to age matched ER+ sporadic breast cancers [20]. Our current findings that ER+ cancers with loss of wt *BRCA1 *are significantly more often higher grade cancers is a consistent extension of our original results. Interestingly, a recent study found that ER+ cancers which develop in *BRCA2 *carriers are of higher grade than age matched ER+ sporadic cancers [40]. Combined with our data, it appears that loss of *BRCA1 *or *BRCA2 *function results in a more proliferative luminal cancer when an ER+ cancer develops.

It has been suggested that basal-like cytokeratin expression in triple negative tumors is a good predictor of *BRCA1 *mutation status [7]. Rakha *et al*. [41] examined seventeen *BRCA1*-associated ER-, HER2- breast cancers and found that only one of seventeen (5.9%) did not show expression of either CK5/6 or EGFR, also considered to be a basal marker. To distinguish between those ER- *BRCA1*-associated breast cancers that did or did not have loss of the wt *BRCA1 *allele, however, we found the combination of CK5/6 and CK14 most useful. Adding EGFR staining increased the sensitivity of identifying ER- cancers with loss of wt *BRCA1 *(28/28; 100%), however it lowered the specificity as three of four of the ER- cancers without loss of wt *BRCA1 *(75%) stained for EGFR.

Immunostains were less helpful in distinguishing ER+ cancers with and without loss of the wt *BRCA1 *allele. It is noteworthy that none of the ER+ cancers that retained wt *BRCA1 *expressed CK5/6 or 14, while the only ER+ cancers expressing these basal cytokeratins had lost wt *BRCA1*. However, as the majority of the ER+ cancers with loss of wt *BRCA1 *did not express either basal cytokeratin, this difference was not significant. Larger studies are necessary to explore the possibility that basal epithelial markers may mark ER+ cancers that have lost wt *BRCA1*.

Our results regarding HER2 overexpression/gene amplification in *BRCA1*-associated cancers are consistent with prior studies that have found that HER2 overexpression and amplification are uncommon in these tumors [5,6,42]. In the 77 *BRCA1 *cancers we studied, only one *BRCA1-*associated cancer with loss of wt *BRCA1 *demonstrated HER2 gene amplification by FISH.

There may be mechanisms other than LOH or methylation by which the wt *BRCA1 *allele is inactivated in *BRCA1*-associated cancers which were not examined in this study (for example, somatic mutation of the second allele elsewhere in the *BRCA1 *gene or ID4 modulation of *BRCA1 *expression [37,43]). However, significant phenotypic differences were observed between ER+ cancers with or without loss of wt *BRCA1*. Assuming phenotype is linked to gene function, these phenotypic differences suggest that ER+ cancers with a wt *BRCA1 *allele are likely to also have retained *BRCA1 *function and have not inactivated wt *BRCA1 *by an alternative mechanism.

It has been previously reported that ER+ breast cancers are more likely to develop in *BRCA1 *carriers as they age, suggesting that some of these may be incidental breast cancers occurring in *BRCA1 *carriers. However, we did not see a significant difference in age at diagnosis between first ER+ breast cancers with and without loss of wt *BRCA1*. In addition all 10 ER+ second cancers that developed in *BRCA1 *carriers demonstrated loss of wt *BRCA1*. It is possible that these findings are due to limited numbers, but it is also possible that some mechanism other than incidental development of breast cancer, with functioning wt *BRCA1*, is needed to explain why the development of ER+ breast cancers is more common as *BRCA1 *mutation carriers age.

Further, it is apparent that the presence of wt *BRCA1 *is not required for ER expression in cancer tissues, in contrast to what has been suggested by some preclinical studies [16]. Other studies have proposed wt *BRCA1 *is essential for differentiation of mammary stem cells to ER+ luminal cells and that loss of wt *BRCA1 *causes an expansion of ER-negative mammary stem cells, offering a mechanism for the common ER-negativity of *BRCA1 *breast cancers [32]. However, this model does not address the origin of ER+ *BRCA1*-associated breast cancers. Another recent study has found expansion of a committed luminal progenitor population, containing both ER+ and ER- cells, in preneoplastic tissues of *BRCA1 *mutation carriers and proposed the luminal progenitor cells as the cell of origin of *BRCA1*-associated cancers [44]. In mouse models of tumorigenesis produced by deletion of *BRCA1*, the expression of ER in the resulting tumors appears to depend on whether *BRCA1 *is deleted at an earlier or later stage of cell differentiation [32,45,46]. Our findings are consistent with these models and suggest that *BRCA1*-deficient ER+ tumors may derive from *BRCA1 *loss in an ER-positive luminal progenitor cell.

This study cannot resolve whether ER+ breast cancers without loss of wt allele that develop in *BRCA1 *carriers are equivalent to ER+ sporadic breast cancers that occur in non-carriers. It is possible that no breast cancer that develops in a *BRCA1 *mutation carrier is really "incidental" or sporadic even if one functioning wt *BRCA1 *allele is retained and expressed. Haploinsufficiency of *BRCA1 *may predispose both to the development of breast cancer as well as to a specific histopathologic and or immunohistochemical profile. However, testing for differences between *BRCA1*-associated ER+ cancers with a retained wt *BRCA1 *and ER+ sporadic breast cancers would require larger numbers of these ER+ *BRCA1 *cancers as well as age matched ER+ sporadic controls.

## Conclusions

In conclusion, in this study of 77 *BRCA1*-associated breast cancers, we found similar frequencies of LOH with loss of wt *BRCA1 *in ER+ and ER- breast cancers. In addition, loss of wt *BRCA1 *results in higher grade ductal cancers with higher proliferative rates, and a greater propensity to express basal cytokeratins. Many of the new therapies being evaluated in *BRCA1 *breast cancers, such as poly(ADP-ribose) polymerase (PARP) inhibitors and cisplatin [47,48], are designed to take advantage of the defect in homologous recombination that *BRCA1 *deficiency causes in these cancers. The results of our study suggest that allele-specific LOH analysis to evaluate for loss of wt *BRCA1 *is more likely to predict response to such therapies than estrogen receptor expression. In addition to family history and age of onset, identification of women who may carry a previously undetected *BRCA1 *mutation has recently focused on the triple negative subset as a population enriched for *BRCA1 *mutation carriers. Our results suggest that the high grade ER+ luminal cancers also may be enriched for tumors with *BRCA1 or BRCA2 *deficiency. A concerted effort should be made to identify these women so that they are not deprived of potentially effective new therapies.

## Abbreviations

CI: confidence interval; CK: cytokeratin; CV: coefficient of variation; ER: estrogen receptor; FISH: fluorescence *in situ *hybridization; HPF: high powered field; IHC: immunohistochemistry; LOH: loss of heterozygosity; m%: mutant *BRCA1 *DNA/mutant plus wt *BRCA1 *DNA; NM_score, normal/mutant allele; OR: odds ratio; PARP: poly(ADP-ribose) polymerase; PCR: polymerase chain reaction; PR: progesterone receptor; TMA: tissue microarray; wt: wild type.

## Competing interests

The authors declare that they have no competing interests.

## Authors' contributions

NT wrote the manuscript (with AM and ALR), shared in study design, oversaw the collection of data and pathology material and reviewed the data. AM shared in the design of the study, performed the LOH analyses and shared in writing the manuscript. SJS shared in study design, reviewed (with LC) all pathology material, reviewed data and helped in editing the manuscript. SG performed statistical analyses; KF served as the study coordinator and data manager. JK aided in collection and review of pathology material as well as analyses of IHC stains. JK, YY, AB, JYK, AS, RT and ZCW performed laser capture microdissection, DNA extraction and methylation studies. LC (with SS) reviewed all study pathology material. KK, RDL, JB, PDR and DS identified cases and supplied pathologic material. DPS helped in study design and manuscript editing. JEG helped in study design, identification of study material, and manuscript editing. ALR co-wrote the manuscript, oversaw microdissections and methylation studies, and shared in study design and data review.

## Supplementary Material

Additional file 1 **Sequence Primers**. Sequences of primers used to amplify the regions surrounding each mutation analyzed in the study are provided. Click here for file

Additional file 2 **Indel Analysis by denaturing capillary electrophoresis**. An example of loss of the wt *BRCA1 *allele (LOHwt) in a tumor from a patient with a 187delAG mutation is provided. Click here for file

Additional file 3 **Supplementary methods**. Additional information describing the methodology used to determine LOH is supplied. Click here for file

Additional file 4 **Distribution of allele loss in tumors with LOH**. This table presents the observed compared to the expected frequencies of loss of the wt and mutant *BRCA1 *alleles in ER+ and ER- *BRCA1 *breast cancers analyzed. Click here for file

Additional file 5 **LOH result for ER+ and ER- *BRCA1*-associated breast cancers analyzed**. For each breast cancer analyzed, the following associated features are presented: specific mutation; age at diagnosis; ER status; m% or NM_score; LOH result; and *BRCA1 *promoter methylation. Click here for file

## References

[B1] Eisinger F Stoppa-Lyonnet D Longy M Kerangueven F Noguchi T Bailly C Vincent-Salomon A Jacquemier J Birnbaum D Sobol H Germ line mutation at BRCA1 affects the histoprognostic grade in hereditary breast cancer Cancer Res 1996 56 471 474 8564955

[B2] Karp SE Tonin PN Begin LR Martinez JJ Zhang JC Pollak MN Foulkes WD Influence of BRCA1 mutations on nuclear grade and estrogen receptor status of breast carcinoma in Ashkenazi Jewish women Cancer 1997 80 435 441 10.1002/(SICI)1097-0142(19970801)80:3<435::AID-CNCR11>3.0.CO;2-Y 9241077

[B3] Robson M Gilewski T Haas B Levin D Borgen P Rajan P Hirschaut Y Pressman P Rosen PP Lesser ML Norton L Offit K BRCA-associated breast cancer in young women J Clin Oncol 1998 16 1642 1649 9586873 10.1200/JCO.1998.16.5.1642

[B4] Verhoog LC Brekelmans CT Seynaeve C van den Bosch LM Dahmen G van Geel AN Tilanus-Linthorst MM Bartels CC Wagner A van den Ouweland A Devilee P Meijers-Heijboer EJ Klijn JG Survival and tumour characteristics of breast-cancer patients with germline mutations of BRCA1 Lancet 1998 351 316 321 10.1016/S0140-6736(97)07065-7 9652611

[B5] Quenneville LA Phillips KA Ozcelik H Parkes RK Knight JA Goodwin PJ Andrulis IL O'Malley FP HER-2/neu status and tumor morphology of invasive breast carcinomas in Ashkenazi women with known BRCA1 mutation status in the Ontario Familial Breast Cancer Registry Cancer 2002 95 2068 2075 10.1002/cncr.10949 12412159

[B6] Lakhani SR Van De Vijver MJ Jacquemier J Anderson TJ Osin PP McGuffog L Easton DF The pathology of familial breast cancer: predictive value of immunohistochemical markers estrogen receptor, progesterone receptor, HER-2, and p53 in patients with mutations in BRCA1 and BRCA2 J Clin Oncol 2002 20 2310 2318 10.1200/JCO.2002.09.023 11981002

[B7] Foulkes WD Stefansson IM Chappuis PO Begin LR Goffin JR Wong N Trudel M Akslen LA Germline BRCA1 mutations and a basal epithelial phenotype in breast cancer J Natl Cancer Inst 2003 95 1482 1485 14519755 10.1093/jnci/djg050

[B8] Atchley DP Albarracin CT Lopez A Valero V Amos CI Gonzalez-Angulo AM Hortobagyi GN Arun BK Clinical and pathologic characteristics of patients with BRCA-positive and BRCA-negative breast cancer J Clin Oncol 2008 26 4282 4288 10.1200/JCO.2008.16.6231 18779615 PMC6366335

[B9] Armes JE Egan AJ Southey MC Dite GS McCredie MR Giles GG Hopper JL Venter DJ The histologic phenotypes of breast carcinoma occurring before age 40 years in women with and without BRCA1 or BRCA2 germline mutations: a population-based study Cancer 1998 83 2335 2345 10.1002/(SICI)1097-0142(19981201)83:11<2335::AID-CNCR13>3.0.CO;2-N 9840533

[B10] Lakhani SR Jacquemier J Sloane JP Gusterson BA Anderson TJ van de Vijver MJ Farid LM Venter D Antoniou A Storfer-Isser A Smyth E Steel CM Haites N Scott RJ Goldgar D Neuhausen S Daly PA Ormiston W McManus R Scherneck S Ponder BA Ford D Peto J Stoppa-Lyonnet D Bignon YJ Struewing JP Spurr NK Bishop DT Klijn JG Devilee P Multifactorial analysis of differences between sporadic breast cancers and cancers involving BRCA1 and BRCA2 mutations J Natl Cancer Inst 1998 90 1138 1145 10.1093/jnci/90.15.1138 9701363

[B11] Laakso M Loman N Borg A Isola J Cytokeratin 5/14-positive breast cancer: true basal phenotype confined to BRCA1 tumors Mod Pathol 2005 18 1321 1328 10.1038/modpathol.3800456 15990899

[B12] Lakhani SR Reis-Filho JS Fulford L Penault-Llorca F van der Vijver M Parry S Bishop T Benitez J Rivas C Bignon YJ Chang-Claude J Hamann U Cornelisse CJ Devilee P Beckmann MW Nestle-Kramling C Daly PA Haites N Varley J Lalloo F Evans G Maugard C Meijers-Heijboer H Klijn JG Olah E Gusterson BA Pilotti S Radice P Scherneck S Sobol H Prediction of BRCA1 status in patients with breast cancer using estrogen receptor and basal phenotype Clin Cancer Res 2005 11 5175 5180 10.1158/1078-0432.CCR-04-2424 16033833

[B13] Arnes JB Brunet JS Stefansson I Begin LR Wong N Chappuis PO Akslen LA Foulkes WD Placental cadherin and the basal epithelial phenotype of BRCA1-related breast cancer Clin Cancer Res 2005 11 4003 4011 10.1158/1078-0432.CCR-04-2064 15930334

[B14] Foulkes WD Metcalfe K Sun P Hanna WM Lynch HT Ghadirian P Tung N Olopade OI Weber BL McLennan J Olivotto IA Begin LR Narod SA Estrogen receptor status in BRCA1- and BRCA2-related breast cancer: the influence of age, grade, and histological type Clin Cancer Res 2004 10 2029 2034 10.1158/1078-0432.CCR-03-1061 15041722

[B15] Harte MT O'Brien GJ Ryan NM Gorski JJ Savage KI Crawford NT Mullan PB Harkin DP BRD7, a subunit of SWI/SNF complexes, binds directly to BRCA1 and regulates BRCA1-dependent transcription Cancer Res 2010 70 2538 2547 10.1158/0008-5472.CAN-09-2089 20215511

[B16] Hosey AM Gorski JJ Murray MM Quinn JE Chung WY Stewart GE James CR Farragher SM Mulligan JM Scott AN Dervan PA Johnston PG Couch FJ Daly PA Kay E McCann A Mullan PB Harkin DP Molecular basis for estrogen receptor alpha deficiency in BRCA1-linked breast cancer J Natl Cancer Inst 2007 99 1683 1694 10.1093/jnci/djm207 18000219 PMC6485437

[B17] Gorski JJ Kennedy RD Hosey AM Harkin DP The complex relationship between BRCA1 and ERalpha in hereditary breast cancer Clin Cancer Res 2009 15 1514 1518 10.1158/1078-0432.CCR-08-0640 19223511 PMC2780737

[B18] Johannsson OT Idvall I Anderson C Borg A Barkardottir RB Egilsson V Olsson H Tumour biological features of BRCA1-induced breast and ovarian cancer Eur J Cancer 1997 33 362 371 10.1016/S0959-8049(97)89007-7 9155518

[B19] Loman N Johannsson O Bendahl PO Borg A Ferno M Olsson H Steroid receptors in hereditary breast carcinomas associated with BRCA1 or BRCA2 mutations or unknown susceptibility genes Cancer 1998 83 310 319 10.1002/(SICI)1097-0142(19980715)83:2<310::AID-CNCR15>3.0.CO;2-W 9669814

[B20] Tung N Wang Y Collins LC Kaplan J Li H Gelman R Comander AH Gallagher B Fetten K Krag K Stoeckert KA Legare RD Sgroi D Ryan PD Garber JE Schnitt SJ Estrogen receptor positive breast cancers in BRCA1 mutation carriers: clinical risk factors and pathologic features Breast Cancer Res 2010 12 R12 10.1186/bcr2478 20149218 PMC2880433

[B21] Tutt A Robson M Garber JE Domchek S Audeh MW Weitzel JN Friedlander M Carmichael J Phase II trial of the oral PARP inhibitor olaparib in BRCA-deficient advanced breast cancer Journal of Clinical Oncology 2009 27 CRA501

[B22] Dworkin AM Spearman AD Tseng SY Sweet K Toland AE Methylation not a frequent "second hit" in tumors with germline BRCA mutations Fam Cancer 2009 8 339 346 10.1007/s10689-009-9240-1 19340607

[B23] Osorio A de la Hoya M Rodriguez-Lopez R Martinez-Ramirez A Cazorla A Granizo JJ Esteller M Rivas C Caldes T Benitez J Loss of heterozygosity analysis at the BRCA loci in tumor samples from patients with familial breast cancer Int J Cancer 2002 99 305 309 10.1002/ijc.10337 11979449

[B24] Manie E Vincent-Salomon A Lehmann-Che J Pierron G Turpin E Warcoin M Gruel N Lebigot I Sastre-Garau X Lidereau R Remenieras A Feunteun J Delattre O de Thé H Stoppa-Lyonnet D Stern MH High frequency of TP53 mutation in BRCA1 and sporadic basal-like carcinomas but not in BRCA1 luminal breast tumors Cancer Res 2009 69 663 671 10.1158/0008-5472.CAN-08-1560 19147582

[B25] Sanger F Nicklen S Coulson AR DNA sequencing with chain-terminating inhibitors Proc Natl Acad Sci USA 1977 74 5463 5467 10.1073/pnas.74.12.5463 271968 PMC431765

[B26] Rozen S Skaletsky HJ Krawetz S, Misener S Primer3 on the WWW for general users and for biologist programmers Bioinformatics Methods and Protocols: Methods in Molecular Biology 2000 Totowa, NJ: Humana Press 365 386 10.1385/1-59259-192-2:365 10547847

[B27] Integrated DNA Technologies http://www.idtdna.com

[B28] Brownie J Shawcross S Theaker J Whitcombe D Ferrie R Newton C Little S The elimination of primer-dimer accumulation in PCR Nucleic Acids Res 1997 25 3235 3241 10.1093/nar/25.16.3235 9241236 PMC146890

[B29] Agencourt protocols: AMPure http://www.beckmangenomics.com/documents/products/ampurexp/AMPureXPProtocol_000387v001.pdf

[B30] Agencourt protocols: CleanSEQ http://www.beckmangenomics.com/documents/products/cleanseq/Agencourt_CleanSEQ_Protocol.pdf

[B31] Matros E Wang ZC Lodeiro G Miron A Iglehart JD Richardson AL BRCA1 promoter methylation in sporadic breast tumors: relationship to gene expression profiles Breast Cancer Res Treat 2005 91 179 186 10.1007/s10549-004-7603-8 15868446

[B32] Liu S Ginestier C Charafe-Jauffret E Foco H Kleer CG Merajver SD Dontu G Wicha MS BRCA1 regulates human mammary stem/progenitor cell fate Proc Natl Acad Sci USA 2008 105 1680 1685 10.1073/pnas.0711613105 18230721 PMC2234204

[B33] Gonzalez R Silva JM Dominguez G Garcia JM Martinez G Vargas J Provencio M Espana P Bonilla F Detection of loss of heterozygosity at RAD51, RAD52, RAD54 and BRCA1 and BRCA2 loci in breast cancer: pathological correlations Br J Cancer 1999 81 503 509 10.1038/sj.bjc.6690722 10507777 PMC2362917

[B34] Wang ZC Lin M Wei LJ Li C Miron A Lodeiro G Harris L Ramaswamy S Tanenbaum DM Meyerson M Iglehart JD Richardson A Loss of heterozygosity and its correlation with expression profiles in subclasses of invasive breast cancers Cancer Res 2004 64 64 71 10.1158/0008-5472.CAN-03-2570 14729609

[B35] Richardson AL Wang ZC De Nicolo A Lu X Brown M Miron A Liao X Iglehart JD Livingston DM Ganesan S X chromosomal abnormalities in basal-like human breast cancer Cancer Cell 2006 9 121 132 10.1016/j.ccr.2006.01.013 16473279

[B36] Suijkerbuijk KP Fackler MJ Sukumar S van Gils CH van Laar T van der Wall E Vooijs M van Diest PJ Methylation is less abundant in BRCA1-associated compared with sporadic breast cancer Ann Oncol 2008 19 1870 1874 10.1093/annonc/mdn409 18647968 PMC2733079

[B37] Turner NC Reis-Filho JS Russell AM Springall RJ Ryder K Steele D Savage K Gillett CE Schmitt FC Ashworth A Tutt AN BRCA1 dysfunction in sporadic basal-like breast cancer Oncogene 2007 26 2126 2132 10.1038/sj.onc.1210014 17016441

[B38] Vasilatos SN Broadwater G Barry WT Baker JC Jr Lem S Dietze EC Bean GR Bryson AD Pilie PG Goldenberg V Skaar D Paisie C Torres-Hernandez A Grant TL Wilke LG Ibarra-Drendall C Ostrander JH D'Amato NC Zalles C Jirtle R Weaver VM Seewaldt VL CpG island tumor suppressor promoter methylation in non-BRCA-associated early mammary carcinogenesis Cancer Epidemiol Biomarkers Prev 2009 18 901 914 10.1158/1055-9965.EPI-08-0875 19258476 PMC2667866

[B39] Kontorovich T Cohen Y Nir U Friedman E Promoter methylation patterns of ATM, ATR, BRCA1, BRCA2 and P53 as putative cancer risk modifiers in Jewish BRCA1/BRCA2 mutation carriers Breast Cancer Res Treat 2009 116 195 200 10.1007/s10549-008-0121-3 18642075

[B40] Bane AL Beck JC Bleiweiss I Buys SS Catalano E Daly MB Giles G Godwin AK Hibshoosh H Hopper JL John EM Layfield L Longacre T Miron A Senie R Southey MC West DW Whittemore AS Wu H Andrulis IL O'Malley FP for the Breast Cancer Family Registry BRCA2 mutation-associated breast cancers exhibit a distinguishing phenotype based on morphology and molecular profiles from tissue microarrays Am J Surg Pathol 2007 31 121 128 10.1097/01.pas.0000213351.49767.0f 17197928

[B41] Rakha EA Elsheikh SE Aleskandarany MA Habashi HO Green AR Powe DG El-Sayed ME Benhasouna A Brunet JS Akslen LA Evans AJ Blamey R Reis-Filho JS Foulkes WD Ellis IO Triple-negative breast cancer: distinguishing between basal and nonbasal subtypes Clin Cancer Res 2009 15 2302 2310 10.1158/1078-0432.CCR-08-2132 19318481

[B42] Palacios J Honrado E Osorio A Cazorla A Sarrio D Barroso A Rodriguez S Cigudosa JC Diez O Alonso C Lerma E Sanchez L Rivas C Benitez J Immunohistochemical characteristics defined by tissue microarray of hereditary breast cancer not attributable to BRCA1 or BRCA2 mutations: differences from breast carcinomas arising in BRCA1 and BRCA2 mutation carriers Clin Cancer Res 2003 9 3606 3614 14506147

[B43] Beger C Pierce LN Kruger M Marcusson EG Robbins JM Welcsh P Welch PJ Welte K King MC Barber JR Wong-Staal F Identification of Id4 as a regulator of BRCA1 expression by using a ribozyme-library-based inverse genomics approach Proc Natl Acad Sci USA 2001 98 130 135 10.1073/pnas.98.1.130 11136250 PMC14556

[B44] Lim E Vaillant F Wu D Forrest NC Pal B Hart AH Asselin-Labat ML Gyorki DE Ward T Partanen A Feleppa F Huschtscha LI Thorne HJ Fox SB Yan M French JD Brown MA Smyth GK Visvader JE Lindeman GJ Aberrant luminal progenitors as the candidate target population for basal tumor development in BRCA1 mutation carriers Nat Med 2009 15 907 913 10.1038/nm.2000 19648928

[B45] Poole AJ Li Y Kim Y Lin SC Lee WH Lee EY Prevention of Brca1-mediated mammary tumorigenesis in mice by a progesterone antagonist Science 2006 314 1467 1470 10.1126/science.1130471 17138902

[B46] Ginestier C Liu S Wicha MS Getting to the root of BRCA1-deficient breast cancer Cell Stem Cell 2009 5 229 230 10.1016/j.stem.2009.08.007 19733528

[B47] Tutt A Robson M Garber JE Domchek SM Audeh MW Weitzel JN Friedlander M Arun B Loman N Schmutzler RK Wardley A Mitchell G Earl H Wickens M Carmichael J Oral poly(ADP-ribose) polymerase inhibitor olaparib in patients with BRCA1 or BRCA2 mutations and advanced breast cancer: a proof-of-concept trial Lancet 2010 376 235 244 10.1016/S0140-6736(10)60892-6 20609467

[B48] Byrski T Huzarski T Dent R Gronwald J Zuziak D Cybulski C Kladny J Gorski B Lubinski J Narod SA Response to neoadjuvant therapy with cisplatin in BRCA1-positive breast cancer patients Breast Cancer Res Treat 2009 115 359 363 10.1007/s10549-008-0128-9 18649131

