# Supplementary material for: Prevalence and predictors of loss of wild type BRCA1 in estrogen receptor positive and negative BRCA1-associated breast cancers
Source: Breast Cancer Res. 2010 Nov 16;12(6):R95. doi: 10.1186/bcr2776 (PMC3046438; doi:10.1186/bcr2776)
Supplement: Additional file 1 — Sequence Primers. Sequences of primers used to amplify the regions surrounding each mutation analyzed in the study are provided. [file bcr2776-S1.ppt]

## Slide 1
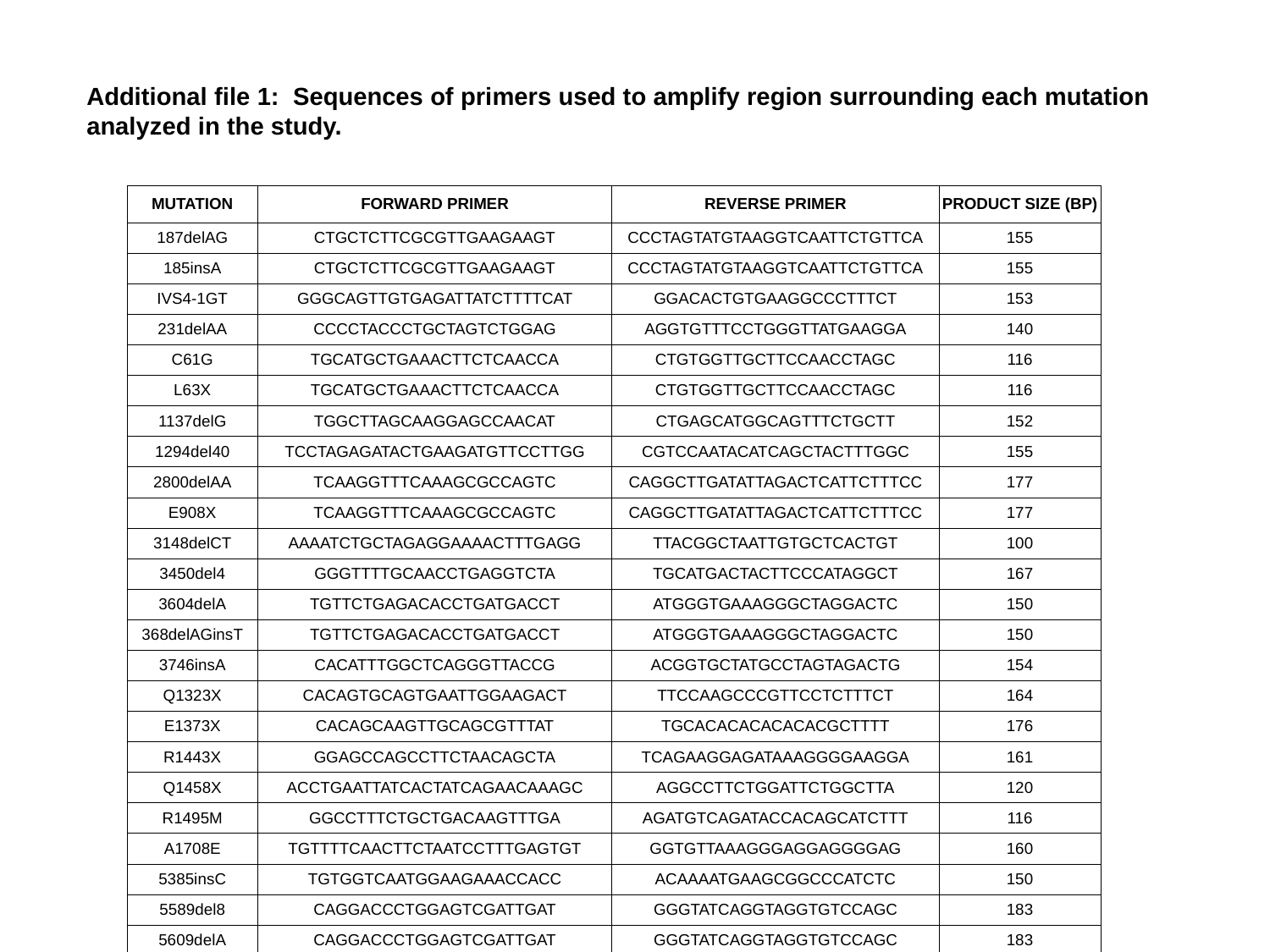

Additional file 1: Sequences of primers used to amplify region surrounding each mutation analyzed in the study.
| MUTATION | FORWARD PRIMER | REVERSE PRIMER | PRODUCT SIZE (BP) |
| --- | --- | --- | --- |
| 187delAG | CTGCTCTTCGCGTTGAAGAAGT | CCCTAGTATGTAAGGTCAATTCTGTTCA | 155 |
| 185insA | CTGCTCTTCGCGTTGAAGAAGT | CCCTAGTATGTAAGGTCAATTCTGTTCA | 155 |
| IVS4-1GT | GGGCAGTTGTGAGATTATCTTTTCAT | GGACACTGTGAAGGCCCTTTCT | 153 |
| 231delAA | CCCCTACCCTGCTAGTCTGGAG | AGGTGTTTCCTGGGTTATGAAGGA | 140 |
| C61G | TGCATGCTGAAACTTCTCAACCA | CTGTGGTTGCTTCCAACCTAGC | 116 |
| L63X | TGCATGCTGAAACTTCTCAACCA | CTGTGGTTGCTTCCAACCTAGC | 116 |
| 1137delG | TGGCTTAGCAAGGAGCCAACAT | CTGAGCATGGCAGTTTCTGCTT | 152 |
| 1294del40 | TCCTAGAGATACTGAAGATGTTCCTTGG | CGTCCAATACATCAGCTACTTTGGC | 155 |
| 2800delAA | TCAAGGTTTCAAAGCGCCAGTC | CAGGCTTGATATTAGACTCATTCTTTCC | 177 |
| E908X | TCAAGGTTTCAAAGCGCCAGTC | CAGGCTTGATATTAGACTCATTCTTTCC | 177 |
| 3148delCT | AAAATCTGCTAGAGGAAAACTTTGAGG | TTACGGCTAATTGTGCTCACTGT | 100 |
| 3450del4 | GGGTTTTGCAACCTGAGGTCTA | TGCATGACTACTTCCCATAGGCT | 167 |
| 3604delA | TGTTCTGAGACACCTGATGACCT | ATGGGTGAAAGGGCTAGGACTC | 150 |
| 368delAGinsT | TGTTCTGAGACACCTGATGACCT | ATGGGTGAAAGGGCTAGGACTC | 150 |
| 3746insA | CACATTTGGCTCAGGGTTACCG | ACGGTGCTATGCCTAGTAGACTG | 154 |
| Q1323X | CACAGTGCAGTGAATTGGAAGACT | TTCCAAGCCCGTTCCTCTTTCT | 164 |
| E1373X | CACAGCAAGTTGCAGCGTTTAT | TGCACACACACACACGCTTTT | 176 |
| R1443X | GGAGCCAGCCTTCTAACAGCTA | TCAGAAGGAGATAAAGGGGAAGGA | 161 |
| Q1458X | ACCTGAATTATCACTATCAGAACAAAGC | AGGCCTTCTGGATTCTGGCTTA | 120 |
| R1495M | GGCCTTTCTGCTGACAAGTTTGA | AGATGTCAGATACCACAGCATCTTT | 116 |
| A1708E | TGTTTTCAACTTCTAATCCTTTGAGTGT | GGTGTTAAAGGGAGGAGGGGAG | 160 |
| 5385insC | TGTGGTCAATGGAAGAAACCACC | ACAAAATGAAGCGGCCCATCTC | 150 |
| 5589del8 | CAGGACCCTGGAGTCGATTGAT | GGGTATCAGGTAGGTGTCCAGC | 183 |
| 5609delA | CAGGACCCTGGAGTCGATTGAT | GGGTATCAGGTAGGTGTCCAGC | 183 |
| R1835X | CAGGACCCTGGAGTCGATTGAT | GGGTATCAGGTAGGTGTCCAGC | 183 |
| W321X | AAGCAAACAGCCTGGCTTAGCA | TTCTCTCACACAGGGGATCAGC | 138 |
| S713X | TGCAACTGGAGCCAAGAAGAGT | TTTTTCTTCTCTTGGAAGGCTAGGA | 172 |
| 2953delGTAinsC | CAGGCTTTCCTGTGGTTGGTC | CAGTTTCGTTGCCTCTGAACTGA | 111 |
| 4154delA | CTGAAAGCCAGGGAGTTGGTCT | CTATAAATAGACTGGGGCAAACACAAA | 157 |
| Y1463X | AGATTTGTTTTCTCATTCCATTTAAAGCAG | TTCTTACCTTTCCACTCCTGGTTCT | 161 |
| 5454delC | AGAGGGCCTGGGTTAAGTATGC | ACAGGGCACCCAATACTTACTGT | 156 |
