# Supplementary material for: Prevalence and predictors of loss of wild type BRCA1 in estrogen receptor positive and negative BRCA1-associated breast cancers
Source: Breast Cancer Res. 2010 Nov 16;12(6):R95. doi: 10.1186/bcr2776 (PMC3046438; doi:10.1186/bcr2776)
Supplement: Additional file 2 — Indel Analysis by denaturing capillary electrophoresis. An example of loss of the wt BRCA1 allele (LOHwt) in a tumor from a patient with a 187delAG mutation is provided. [file bcr2776-S2.ppt]

## Slide 1
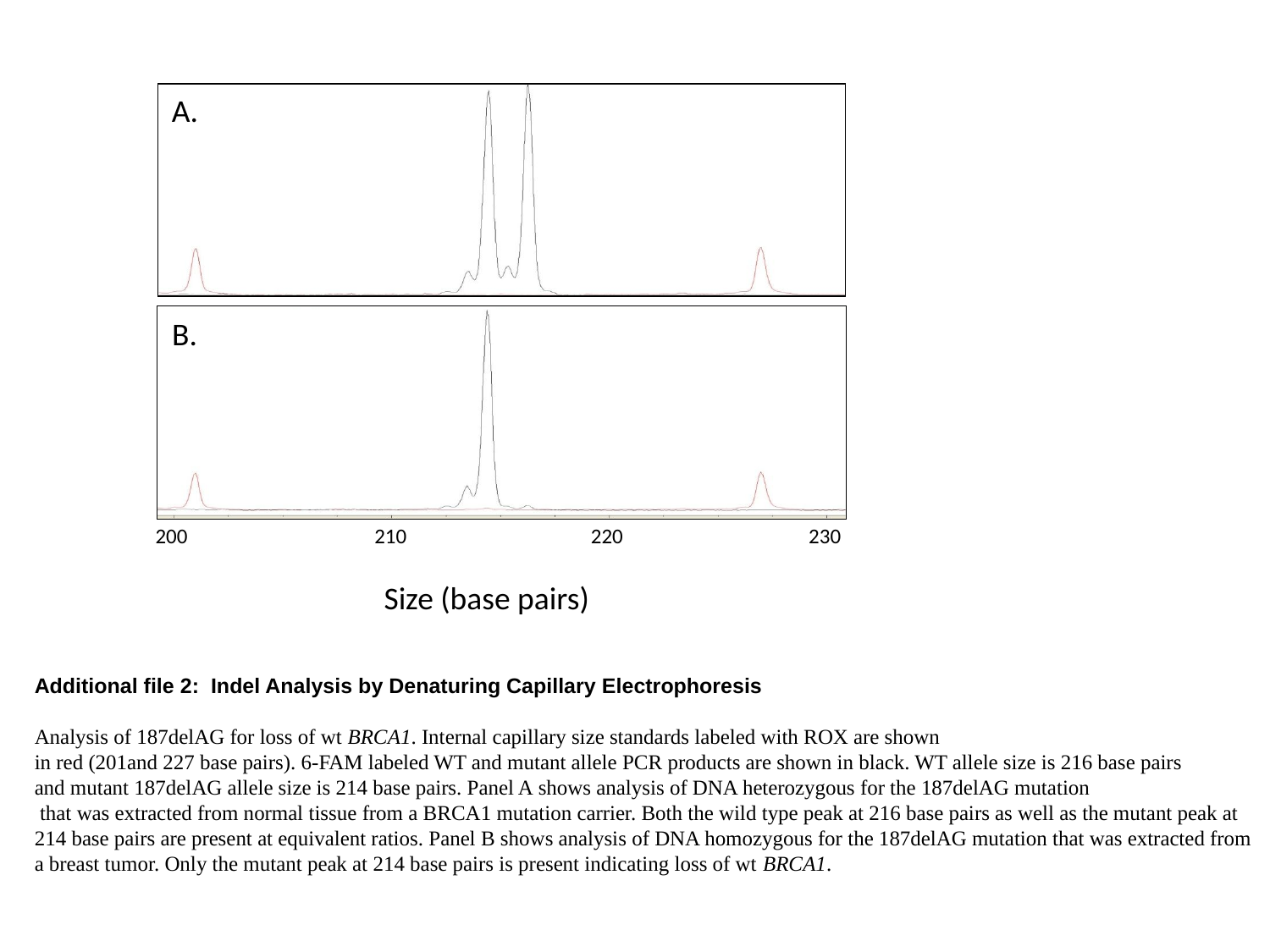

A.
B.
200
210
220
230
Size (base pairs)
Additional file 2: Indel Analysis by Denaturing Capillary Electrophoresis
Analysis of 187delAG for loss of wt BRCA1. Internal capillary size standards labeled with ROX are shown
in red (201and 227 base pairs). 6-FAM labeled WT and mutant allele PCR products are shown in black. WT allele size is 216 base pairs
and mutant 187delAG allele size is 214 base pairs. Panel A shows analysis of DNA heterozygous for the 187delAG mutation
 that was extracted from normal tissue from a BRCA1 mutation carrier. Both the wild type peak at 216 base pairs as well as the mutant peak at 214 base pairs are present at equivalent ratios. Panel B shows analysis of DNA homozygous for the 187delAG mutation that was extracted from a breast tumor. Only the mutant peak at 214 base pairs is present indicating loss of wt BRCA1.
