# Supplementary material for: Prevalence and predictors of loss of wild type BRCA1 in estrogen receptor positive and negative BRCA1-associated breast cancers
Source: Breast Cancer Res. 2010 Nov 16;12(6):R95. doi: 10.1186/bcr2776 (PMC3046438; doi:10.1186/bcr2776)
Supplement: Additional file 3 — Supplementary methods. Additional information describing the methodology used to determine LOH is supplied. [file bcr2776-S3.doc]

**Additional file 3: Supplementary methods**

**LOH analysis**

Screening for LOH was carried out by polymerase chain reaction followed by Sanger dideoxy sequencing [25] for missense and nonsense alterations while denaturing capillary analysis was performed for insertions and deletions. Exon specific oligonucleotide primers flanking known BRCA1 mutations were designed using Primer3 software [26] and limited to a maximum product size of 200 base pairs (sequences listed in Additional file 1). PCR primers were ordered [27] with universal sequence tags at the 5’ end: 5’GCGTACCACGTGTCGACT 3’ (Forward) and 5’GACGGGCGT ACTAGCGTA 3’ (Reverse) in order to allow for secondary amplification, sequencing with a universal primer and elimination of primer dimers [28]. Primary PCR amplification was performed with the exon specific tagged primers in 4uL reaction volume containing 1 ng template DNA, 0.08 U Klear Taq (kbiosciences, UK), 10 mM KCl, 10 mM (NH_4_)_2_SO_4_, 20 mM Tris-HCl (pH 8.8), 2 mM MgSO_4_, 0.2 mM each dNTP and 0.4 uM each primer. Cycling conditions were: 95°C, 15 min enzyme activation followed by 35 cycles of 95°C, 30 s denaturation; 62 °C, 30 s annealing; 68°C, 90 s extension; and a final extension of 72°C for 5 min. In order to get sufficient template material for cycle sequencing, primary PCR products were re-amplified by tip touching into fresh master mix with the same ingredients as above except, instead of the exon specific, universal primer pairs were used. Secondary PCR products were purified from unincorporated primers, dNTPs, DNA polymerases and salts using Agencourt AMPure Kit [29].

Bidirectional DNA sequencing in triplicate was performed using BigDye Terminator v3.1 Cycle Sequencing Kit (Applied Biosystems) and universal primers. Sequencing reactions contained template DNA (purified secondary PCR product), deoxynucleotides for extension from a single primer, fluorescent labeled chain terminators (dideoxynucleotides), sequencing buffer (Applied Biosystems) and DMSO. Unincorporated nucleotides and dye-labeled chain terminators were removed using Agencourt CleanSEQ kits [30]. Purified sequencing products were analyzed in 3730xl DNA Analyzers (Applied Biosystems).

Analysis of LOH in tumors with insertions or deletions in BRCA1 was performed by taking advantage of the difference in size of the wild type and mutant alleles. Primary amplification was performed using the same conditions described for sequencing analysis above. Secondary PCR amplification was used to add a 6-FAM fluorescent label onto the 5’-end of the primary PCR products using labeled primers complementary to the universal primer tag used in primary amplification (5’ 6-FAM-Gtgtatgcgtactagcgtaccacgtgtcgact-3’, 5’-Gtttctgacgatacgacgggcgtactagcgta-3’). This results in PCR products that were extended by a total of 62 base pairs from the predicted (non-tailed) product size. Reaction conditions used for secondary amplification were identical to primary amplification. Reaction products were then analyzed on ABI3730XL (Applied Biosystems, CA) instruments using POP7 denaturing polymer (Applied Biosystems, CA).
