# Supplementary material for: Prevalence and predictors of loss of wild type BRCA1 in estrogen receptor positive and negative BRCA1-associated breast cancers
Source: Breast Cancer Res. 2010 Nov 16;12(6):R95. doi: 10.1186/bcr2776 (PMC3046438; doi:10.1186/bcr2776)
Supplement: Additional file 4 — Distribution of allele loss in tumors with LOH. This table presents the observed compared to the expected frequencies of loss of the wt and mutant BRCA1 alleles in ER+ and ER- BRCA1 breast cancers analyzed. [file bcr2776-S4.pdf]

Additional file 4. Distribution of allele loss in tumors with LOH

	Expected		Observed		Chi-Square p value
	LOH^{mut}	LOH^{wt}	LOH^{mut}	LOH^{wt}	
All tumors with LOH (n=72)	36	36	7	65	< 0.001
ER- tumors with LOH (n=34)	17	17	3	31	< 0.001
ER+ tumors with LOH (n=38)	19	19	4	34	< 0.001
