# Supplementary material for: Prevalence and predictors of loss of wild type BRCA1 in estrogen receptor positive and negative BRCA1-associated breast cancers
Source: Breast Cancer Res. 2010 Nov 16;12(6):R95. doi: 10.1186/bcr2776 (PMC3046438; doi:10.1186/bcr2776)
Supplement: Additional file 5 — LOH result for ER+ and ER- BRCA1-associated breast cancers analyzed. For each breast cancer analyzed, the following associated features are presented: specific mutation; age at diagnosis; ER status; m% or NM_score; LOH result; and BRCA1 promoter methylation. [file bcr2776-S5.pdf]

Additional file 5: LOH result for ER+ and ER- BRCA1-associated breast cancers analyzed.

Patient	Mutation designation	Type of mutation	Age	ER status	m%	NM_score	LOH result	BRCA1 promoter methylation
1	3604delA	deletion	40	Neg	74.7		LOH wt	
2	1137delAG	deletion	35	Neg	82.9		LOH wt	
3	1137delG	deletion	36	Neg	19.3		LOH mut	Neg
4	1294del40	deletion	41	Neg	89.3		LOH wt	Neg
5	1294del40	deletion	44	Pos	91.9		LOH wt	
6	1294del40	deletion	52	Pos	97		LOH wt	
7	1294del40	deletion	32	Pos	98.2		LOH wt	
8	185insA	insertion	40	Pos	39.5		LOH mut	
9	187delAG	deletion	56	Neg	21.7		LOH mut	Neg
10	187delAG	deletion	31	Neg	40.1		no LOH	Neg
11	187delAG	deletion	28	Neg	62.7		LOH wt	
12	187delAG	deletion	44	Neg	65.1		LOH wt	Neg
13	187delAG	deletion	39	Neg	67.4		LOH wt	
14	187delAG	deletion	73	Neg	84.5		LOH wt	
15	187delAG	deletion	44	Neg	91		LOH wt	Neg
16	187delAG	deletion	53	Neg	97.2		LOH wt	Neg
17	187delAG	deletion	54	Neg	97.9		LOH wt	
18	187delAG	deletion	41	Neg	98.5		LOH wt	
19	187delAG	deletion	55	Neg	99.2		LOH wt	
20	187delAG	deletion	72	Pos	44		no LOH	
21	187delAG	deletion	47	Pos	67.3		LOH wt	
22	187delAG	deletion	43	Pos	82.3		LOH wt	Neg
23	187delAG	deletion	50	Pos	86.7		LOH wt	
24	187delAG	deletion	42	Pos	88.4		LOH wt	
25	187delAG	deletion	52	Pos	88.6		LOH wt	
26	187delAG	deletion	34	Pos	89.4		LOH wt	Neg
27	187delAG	deletion	56	Pos	89.8		LOH wt	
28	187delAG	deletion	29	Pos	93.5		LOH wt	
29	187delAG	deletion	45	Pos	93.8		LOH wt	Neg
30	187delAG	deletion	55	Pos	93.9		LOH wt	
31	187delAG	deletion	51	Pos	94.9		LOH wt	
32	187delAG	deletion	68	Pos	95.2		LOH wt	
33	187delAG	deletion	39	Pos	96.1		LOH wt	Neg
34	187delAG	deletion	43	Pos	96.1		LOH wt	
35	187delAG	deletion	51	Pos	98.5		LOH wt	
36	231delAA	deletion	29	Neg	98.3		LOH wt	
37	2800delAA	deletion	44	Neg	97.4		LOH wt	
38	2953delGTAinsC	insertion/deletion	45	Pos	83		LOH wt	Neg
39	3148delCT	deletion	35	Neg	99.2		LOH wt	
40	3450del4	deletion	48	Pos	89.3		LOH wt	
41	3668delAGinsT	insertion/deletion	39	Pos	72.3		LOH wt	Neg
42	3746insA	insertion	46	Neg	91		LOH wt	
43	3790ins4	insertion	49	Pos	5.4		LOH mut	Neg
44	4154delA	deletion	63	Pos	97		LOH wt	
45	5385insC	insertion	58	Neg	3		LOH mut	
46	5385insC	insertion	61	Neg	62.7		LOH wt	
47	5385insC	insertion	45	Neg	77.8		LOH wt	
48	5385insC	insertion	50	Neg	80		LOH wt	Neg
49	5385insC	insertion	44	Neg	80.1		LOH wt	
50	5385insC	insertion	32	Neg	85.5		LOH wt	Neg
51	5385insC	insertion	53	Neg	90.4		LOH wt	
52	5385insC	insertion	42	Neg	90.9		LOH wt	Neg
53	5385insC	insertion	32	Neg	91		LOH wt	
54	5385insC	insertion	45	Pos	27.1		LOH mut	Neg
55	5385insC	insertion	64	Pos	45.3		no LOH	Neg
56	5385insC	insertion	39	Pos	60.7		no LOH	Neg
57	5385insC	insertion	51	Pos	82.3		LOH wt	
58	5385insC	insertion	34	Pos	83.9		LOH wt	Neg
59	5385insC	insertion	46	Pos	88.5		LOH wt	
60	5385insC	insertion	37	Pos	89.7		LOH wt	
61	5385insC	insertion	46	Pos	90		LOH wt	
62	5454delC	deletion	43	Pos	88.7		LOH wt	Neg
63	5589del8	deletion	37	Neg	80.7		LOH wt	
64	A1708E	missense	66	Neg		0.19	LOH wt	
65	C61G	missense	37	Neg		0.31	LOH wt	Neg
66	E908X	nonsense	41	Pos		0.11	LOH wt	
67	IVS4-1G>T	splicing	33	Pos		0.96	no LOH	Neg
68	IVS4-1G>T	splicing	59	Pos		3.05	LOH mut	Pos
69	IVS4-1G>T	splicing	32	Neg		0.23	LOH wt	Neg
70	Q1323X	nonsense	31	Neg		0.05	LOH wt	Neg
71	Q1458X	nonsense	39	Neg		0.27	LOH wt	
72	Q1458X	nonsense	52	Pos		0.32	LOH wt	
73	R1495M	missense	42	Neg		0.48	LOH wt	
74	R1495M	missense	51	Pos		0.1	LOH wt	Neg
75	S713X	nonsense	49	Pos		0.02	LOH wt	
76	W321X	nonsense	47	Pos		0.08	LOH wt	
77	Y1463X	nonsense	46	Pos		0.13	LOH wt	
